# Genistein induces G2/M cell cycle arrest and apoptosis via ATM/p53-dependent pathway in human colon cancer cells

**DOI:** 10.3892/ijo.2013.1946

**Published:** 2013-05-17

**Authors:** ZHIYU ZHANG, CHONG-ZHI WANG, GUANG-JIAN DU, LIAN-WEN QI, TYLER CALWAY, TONG-CHUAN HE, WEI DU, CHUN-SU YUAN

**Affiliations:** 1 Tang Center for Herbal Medicine Research, University of Chicago, Chicago, IL 60637, USA; 2 Department of Anesthesia and Critical Care, University of Chicago, Chicago, IL 60637, USA; 3 Department of Surgery, University of Chicago, Chicago, IL 60637, USA; 4 Ben May Department for Cancer Research, University of Chicago, Chicago, IL 60637, USA; 5 Committee on Clinical Pharmacology and Pharmacogenomics, The Pritzker School of Medicine, University of Chicago, Chicago, IL 60637, USA

**Keywords:** colon cancer, cancer chemoprevention, G2/M cell cycle arrest, isoflavones, p53

## Abstract

Soybean isoflavones have been used as a potential preventive agent in anticancer research for many years. Genistein is one of the most active flavonoids in soybeans. Accumulating evidence suggests that genistein alters a variety of biological processes in estrogen-related malignancies, such as breast and prostate cancers. However, the molecular mechanism of genistein in the prevention of human colon cancer remains unclear. Here we attempted to elucidate the anticarcinogenic mechanism of genistein in human colon cancer cells. First we evaluated the growth inhibitory effect of genistein and two other isoflavones, daidzein and biochanin A, on HCT-116 and SW-480 human colon cancer cells. In addition, flow cytometry was performed to observe the morphological changes in HCT-116/SW-480 cells undergoing apoptosis or cell cycle arrest, which had been visualized using Annexin V-FITC and/or propidium iodide staining. Real-time PCR and western blot analyses were also employed to study the changes in expression of several important genes associated with cell cycle regulation. Our data showed that genistein, daidzein and biochanin A exhibited growth inhibitory effects on HCT-116/SW-480 colon cancer cells and promoted apoptosis. Genistein showed a significantly greater effect than the other two compounds, in a time- and dose-dependent manner. In addition, genistein caused cell cycle arrest in the G2/M phase, which was accompanied by activation of ATM/p53, p21*^waf1/cip1^* and GADD45α as well as downregulation of cdc2 and cdc25A demonstrated by q-PCR and immunoblotting assay. Interestingly, genistein induced G2/M cell cycle arrest in a p53-dependent manner. These findings exemplify that isoflavones, especially genistein, could promote colon cancer cell growth inhibition and facilitate apoptosis and cell cycle arrest in the G2/M phase. The ATM/p53-p21 cross-regulatory network may play a crucial role in mediating the anticarcinogenic activities of genistein in colon cancer.

## Introduction

Colon cancer is one of the most common malignancies and a leading cause of cancer deaths for both men and women worldwide (1,2). Even today its prevalence is rising and the 5-year survival rate is still poor. Although significant advances in diagnosis and therapy have been achieved over the last half century, it remains the second leading cause of cancer deaths among American men and the third among American women (3). Nearly 50% of patients with advanced tumors develop recurrent disease and die soon afterward (4). More powerful and safer chemopreventive or chemotherapeutic approaches are urgently needed to reduce mortality and garner better curative effects, since a large number of patients with advanced disease fail to effectively respond to current treatment regimens (5). In this regard, dietary supplements that are capable of preventing carcinogenesis and inhibiting the growth of colon carcinoma cells have generated intense interest (6).

Epidemiologic studies suggest that dietary factors are one of the major concerns in cancer etiology and may account for ≤35% of the difference in cancer incidences (7,8). For instance, in some countries, such as China, where the consumption of soy isoflavone-containing foods is substantially higher than in western countries, the risk of developing breast cancer has been lower (9). Recent data have also shown that continuous consumption of soy products is also positively correlated with a reduction in other human malignancies, such as prostate, stomach, colorectal and lung cancers (9). Most isoflavones are present in a glycosidic form in nature and can be converted to a corresponding aglycon form under certain conditions (10). Genistein and daidzein, which are aglycon isoflavones, have already been tested as potential cancer preventative agents. Genistein is currently considered to be the primary anticancer component from soybeans, based on its dependable inhibitory effect on tumors compared to daidzein. *In vitro* and *in vivo* studies showed that genistein suppressed angiogenesis and induced apoptosis and cell differentiation by inhibiting protein tyrosine phosphorylation and topoisomerase activity, implying that genistein could be a potential cancer chemopreventive agent (11).

Cancer cells lack normal growth controls, exhibit loss of cell cycle control, have unlimited reproductive potential and have growth-signal self-sufficiency (12). Any compound aimed at controlling these processes would be beneficial in suppressing the progression of tumors. Epigenetic studies have confirmed that cell overproliferation and loss of normal cell cycle regulation are involved in colon cancer growth and progression (13). Current studies have shown that a complicated cluster of regulatory factors, such as extracellular signal-regulated kinases (ERKs), cell cycle regulators and the tumor suppressor gene p53, play a pivotal role in the process of colon cancer progression (13,14). Substantial research is focused on exploring novel compounds that can regulate cell proliferation, cell cycle progression and apoptosis in order to elucidate new candidates for cancer therapy (15). The inhibitory effect of genistein on carcinogenesis and tumor growth has been known for years, but the clear molecular mechanism is still not fully understood. The involvement of estrogen receptors (ERs), tyrosine kinases and the oxidative and angiogenesis pathways have been reported (16,17), providing some insight into the development of the mechanism of the anticancer effect derived from genistein. In the present study, we investigated three isoflavones: genistein, daidzein and biochanin A, to identify a safer, more effective and reliable candidate compound for colon tumor therapy. We examined the effects of each compound on HCT-116 and SW-480 cell growth, apoptosis and cell cycle arrest. Although p53 was regarded as an important defense mechanism that regulates apoptosis and cell cycle arrest during multiple tumor development (18), reports related to the interaction of p53 and genistein in cell cycle controlling are rare. Here we hypothesized that activation of p53/ATM-p21, which is induced by genistein treatment, plays a critical role in the modulation of apoptosis and cell cycle arrest and helps to elucidate the molecular mechanism. Our data demonstrated that genistein induced specific G2/M cell cycle arrest via p53-dependent way by the ATM/p53-p21 cross-talk regulatory pathway, which provided novel evidence in the colon cancer chemoprevention of natural flavones.

## Materials and methods

### Chemicals and reagents

Genistein, daidzein and biochanin A were obtained from Sigma-Aldrich (St. Louis, MO), diluted to 2.5, 5, 10, 25, 50 and 100 mM in DMSO (Fisher Chemicals, Fair Lawn, NJ) and stored in small aliquots at −20°C.

### Cell culture conditions

Human colon cancer cell lines HCT-116 and SW-480 were obtained from the American Type Tissue Collection (Rockville, MD) and maintained in McCoy’s 5A or L-15 medium (Hyclone, Logan, UT). HCT-116 (p53^+/+^) and HCT-116 (p53^−/−^) cells were manipulated and maintained in McCoy’s 5A medium as previously described (19). All media were supplemented with 10% fetal bovine serum (FBS), penicillin (100 IU/ml) and streptomycin (100 *μ*g/ml). The cells were seeded twice a week and incubated at 37°C, 95% humidity, 5% CO_2_.

### MTS assays

For cell proliferation assays, the HCT-116 and SW-480 cells were seeded in 96-well plates at a concentration of 5000/well, allowed to adhere for 24 h and subsequently exposed to different concentrations of compounds (2.5, 5, 10, 25, 50 and 100 *μ*M). After 24, 48 and 72 h, survival and growth were measured by the CellTiter 96 Aqueous MTS Reagent (Promega, Madison, WI) according to the manufacturer’s instructions. The absorbance value was measured by an automatic microplate reader (Epoch; Bio-Tek Instruments, Winooski, VT) at 490 nm. Results are expressed as a percentage versus control (vehicle set at 100%).

### Apoptosis assay

HCT-116 and SW-480 cells were seeded in 24-well plates. After 24 h, the medium was changed and chemicals were added with indicated concentrations. After treatment for 48 h, all the adherent cells were collected with 0.05% trypsin, including the floating cells in the medium. Annexin-V-(FITC) and propidium iodide (PI, Becton-Dickinson, San Diego, CA) were used for staining according to the manufacturer’s instructions. Vehicle-treated cells were set for control. The double-stained cells were subsequently analyzed by a FACSCanto flow cytometer (Becton-Dickinson, Mountain View, CA). All experiments were processed independently three times. At least 10,000 cells were counted each time.

### Cell cycle assay

For cell cycle arrest analysis, HCT-116, SW-480, HCT-116 p53^+/+^ or p53^−/−^ cells were seeded in a 12-well plate. On the second day, the compounds were administered at different concentrations. After 48 h, cells were dispensed and fixed with 80% ethanol and frozen for >2 h at −20°C. With a treatment of 0.25% Triton X-100 for 5 min, the cells were resuspended in 150 *μ*l of PI/RNase staining buffer (Becton-Dickinson, San Diego, CA), incubated in the dark for 20 min, followed by counting with a FACSCanto flow cytometer. At least 10,000 cells were collected for each measurement in a triplicate experiment.

### Real-time PCR array of human cell cycle related genes

Total RNA was extracted 48 h after exposure to genistein using RNeasy mini kit (Qiagen, Valencia, CA) and quantified by Nanodrop (Thermo, Wilmington, DE). The cDNA was created with RT^2^ first strand kit (SAbioscience, Frederick, MD). Then the first strand of transcription product was applied as a template by using Human cell cycle RT2 Profiler PCR array plate (cat no. PAHS-020E, 84 genes covered, SAbioscience) following the manufacturer’s instructions. Experiments were run three times. Relative genes expression quantification was determined using the 2^−ΔΔct^ method.

### Immunoblot assay

The HCT-116 p53^+/+^ and p53^−/−^ cells were lysed in ice-cold radio immunoprecipitation assay (RIPA) buffer supplemented with 1% (v/v) protease inhibitor cocktail and PMSF. Then the lysates were collected and the clear supernatant was stored in aliquots at −80°C for further analysis. The protein concentration of the lysates was determined by a BCA protein assay kit (Pierce, Rockford, IL). Aliquots of the lysates (50 *μ*g of total protein) were denatured with loading buffer for 5 min at 95°C and resolved by 4–15% Mini-PROTEAN TGX precast gel (Bio-Rad, Hercules, CA). The assorted proteins were transferred to a PVDF membrane (Bio-Rad) and blocked in PBST buffer (PBS with 0.05% Tween-20) containing 5% non-fat dried milk. The transferred blots were incubated with various primary antibodies (p53, p21*^waf1/cip1^*, GADD45α, ATM, cdc2, cdc25A and β-actin from Cell Signaling, Danvers, MA) overnight at 4°C, followed by 1-h incubation with appropriate secondary antibodies conjugated to horseradish peroxidase. β-actin expression was used as the loading control. The intensity of the specific immunoreactive bands was detected by SuperSignal West Pico Substrate (Thermo-pierce, Rockford, IL) and quantified by densitometry using ImageJ 1.45 software. Data were supplied as a ratio of the β-actin for analyzing and plotting.

### Statistical analysis

Data are presented as mean ± standard deviation. Comparisons of 2 groups were made by Student’s t-test. A p=0.05 was used to determine significant differences. All analyses were performed using SPSS 14.0 (IBM Corp., Somers, NY).

## Results

### Genistein, daidzein and biochanin A inhibits HCT-116/SW-480 cell proliferation

**Figure 1.**
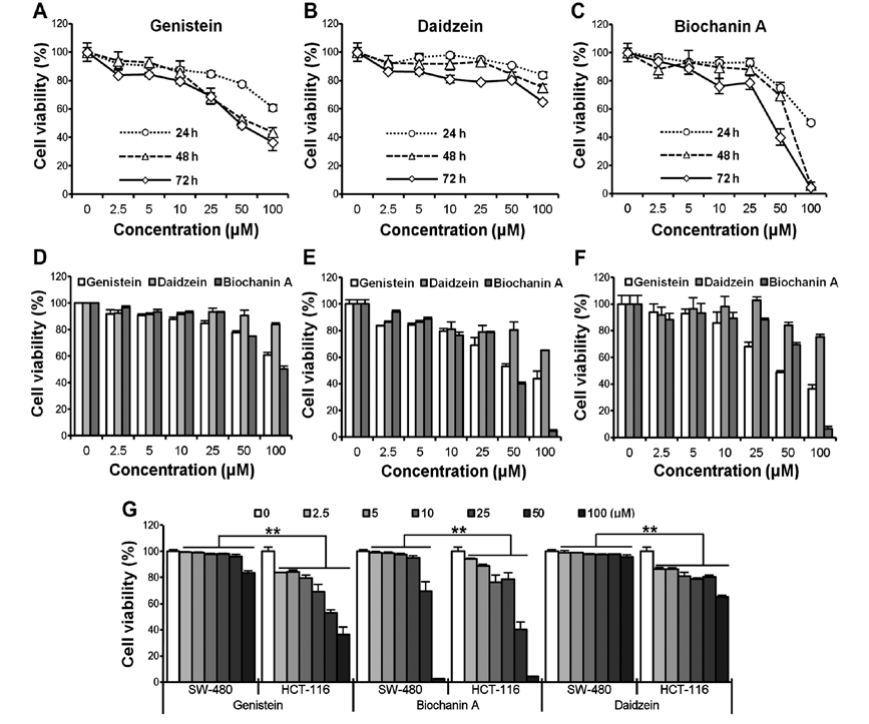
Effect of genistein, daidzein and biochanin A on HCT-116/SW-480 human colon cancer cell proliferation. Cell survival was determined by the MTS assay and calculated as a ratio, comparing it to the vehicle-treated cells. (A–C) HCT-116 cell growth was inhibited in a time-dependent manner 24, 48 and 72 h after genistein, daidzein and biochanin A exposure. (D–F) Dose-dependent response in HCT-116 cells after compounds were treated at 24, 48 and 72 h. (G) Bar plot of HCT-116/SW-480 cells treated with genistein, daidzein or biochanin A at 48 h. HCT-116 cells showed more sensitivtity than SW-480 cells (^**^p<0.01). Data are presented as mean ± SD from at least triplicate wells and 3 independent experiments.

To investigate the growth inhibitory effect of genistein, daidzein and biochanin A, HCT-116/SW-480 cells were treated with different concentrations (2.5, 5, 10, 25, 50, 100 *μ*M) of compounds for different periods of time (24, 48 and 72 h). Cell viability was assessed by the MTS assay. As shown in Fig. 1A–C, the viability of HCT-116 cells decreased at indicated time points in a time-dependent manner. However, cell growth inhibition caused by genistein was relatively remarkable compared to the other two compounds at the logical doses (<50 *μ*M, IC_50_ of genistein and biochanin A were ∼50 *μ*M, HCT-116 cells were more resistant to daidzein compared to the other two compounds). In addition, HCT-116 also acted in a clearly dose-dependent way after genistein administration at 24, 48 and 72 h (Fig. 1D–F). To further compare the cytotoxicity induced by the compounds on cells with different original p53 status, HCT-116 (p53 wild-type) and SW-480 (p53 mutated) cells were simultaneously exposed to the compounds. The results are presented in Fig. 1G. HCT-116 cells were more sensitive than SW-480 cells among the three compounds, suggesting that the p53 status might contribute to the different outcomes after the exposure (^**^p<0.01).

### Genistein, biochanin A and daidzein promote apoptosis

**Figure 2.**
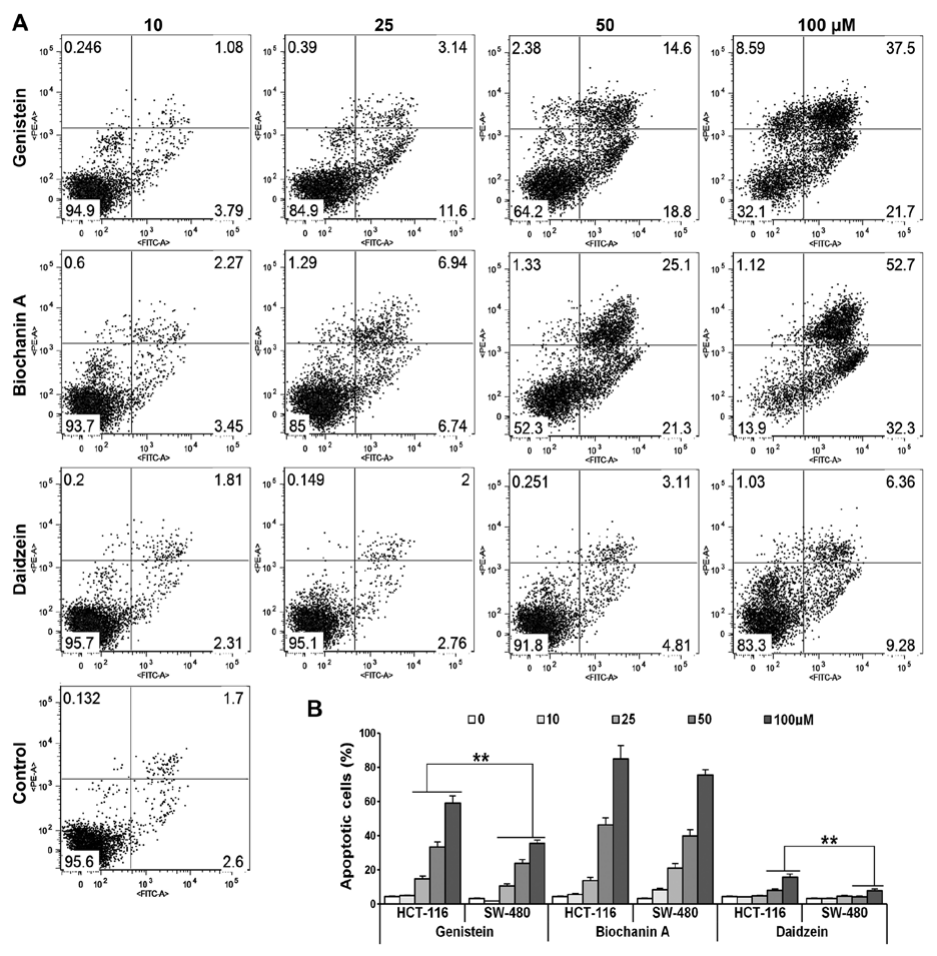
Genistein, biochanin A and daidzein promoted apoptosis in HCT-116/SW-480 cells. Data are counted as a ratio of total cells counted. (A) All compounds induced significant apoptosis in HCT-116 cells. Biochanin A was the most potent proapoptotic chemical among the compounds. (B) Genistein, biochanin A and daidzein induced more apoptotic cells in HCT-116 cells than in SW-480 cells (^**^p<0.01 compared with each other).

HCT-116/SW-480 cells were treated with different concentrations (10, 25, 50 and 100 *μ*M) of genistein, daidzein and biochanin A for 48 h. Apoptotic cells were determined by flow cytometry using Annexin V/propidium iodide (PI) double labeling. The ratio of the apoptotic cells (Annexin V-FITC-positive) in HCT-116 was significantly increased in a dose-dependent manner when treated with genistein or biochanin A. However, no explicit effects were observed after daidzein exposure (Fig. 2A). Biochanin A showed a relatively more intense proapoptotic effect than genistein at higher concentrations (total apoptotic cell ratio 46.4 vs. 33.4% at 50 *μ*M, 85 vs. 59.2% at 100 *μ*M). Compared with the proapoptotic impact promoted by these compounds in SW-480 cells it also showed that HCT-116 cells were more sensitive when exposed to genistein than SW-480 cells (Fig. 2B, ^**^p<0.01), which suggested that the p53 status is crucial for stimulating programmed cell death after genistein administration.

### Genistein, biochanin A and daidzein induce cell cycle arrest in HCT-116/SW-480 cells

**Figure 3.**
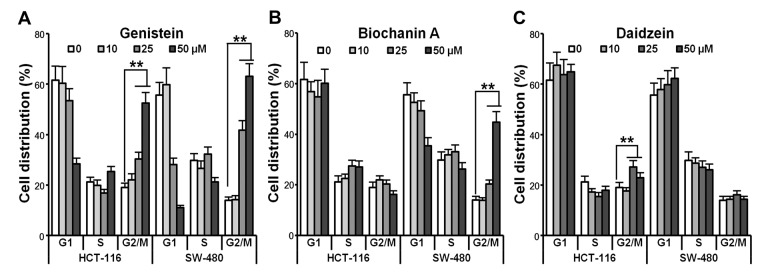
Effect of genistein, biochanin A and daidzein on cell cycle arrest in HCT-116/SW-480 cells. Data are presented as a ratio of total cells counted. (A) Genistein induces G2/M cell cycle arrest in both cell lines at higher doses (50 and 100 *μ*M) in a dose-dependent way 48 h (^**^p<0.01). (B) Biochanin A induces G2/M cell cycle arrest in SW-480 cells at doses (50 and 100 *μ*M) (^**^p<0.01). (C) Daidzein arrests HCT-116 cells at G2/M phase at higher doses (50 and 100 *μ*M) (^**^p<0.01).

The cell cycle assay was also examined by flow cytometry. HCT-116/SW-480 cells were treated with 10, 25, 50 *μ*M of the compounds for 48 h. As shown in Fig. 3A, genistein induced G2/M cell cycle arrest in a dose-dependent manner in both HCT-116 and SW-480 cells (p<0.01). Biochanin A showed an interesting result in that it only expressed G2/M arrest in SW-480 cells but not in HCT-116 cells (Fig. 3B, p<0.01). Daidzein did not have a strong effect in arresting cell progression at the G2/M phase in HCT-116 cells. Due to the above effects induced by genistein, it was selected as a typical isoflavone for use in later experiments.

### Genistein induces genes expression involved in G2 to M transition by RT-PCR

**Figure 4.**
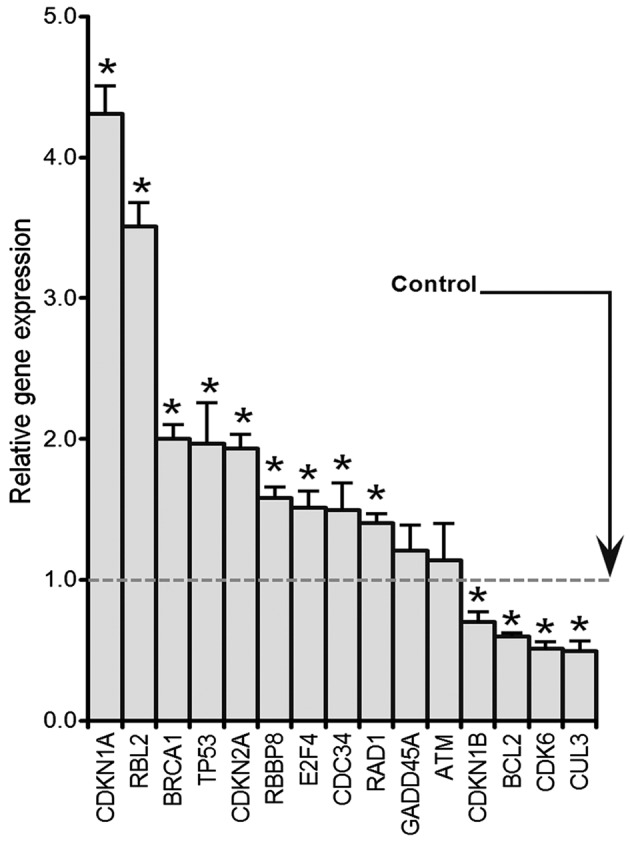
Relative expression of selected cell cycle arrest related genes examined by qPCR array. A human cell cycle RT^2^ Profiler PCR array plate was used to assess the expression of cell cycle related genes. Representative upregulated and downregulated genes are shown. Data are expressed as mean ± SD, relative gene expression was normalized using β-actin as a control and calculated by the 2^−ΔΔct^ method (^*^p<0.05).

HCT-116 cells were treated with 50 *μ*M genistein for 48 h. A human cell cycle RT2 Profiler PCR array kit (cat no. PAHS-020E, 84 genes contained, SAbioscience) was used to determine the expression levels of cell cycle-related genes. Selected genes are presented in Fig. 4 that were either up or downregulated. p53, p21(CDKN1A), BRCA1 and E2F4 were significantly increased compared with the control (p<0.05). In contrast, Bcl2, CDK6 and CUL3 levels were significantly decreased after genistein exposure (p<0.05).

### Genistein induces G2/M cell cycle arrest partially in p53-dependent manner in HCT-116 cells

**Figure 5.**
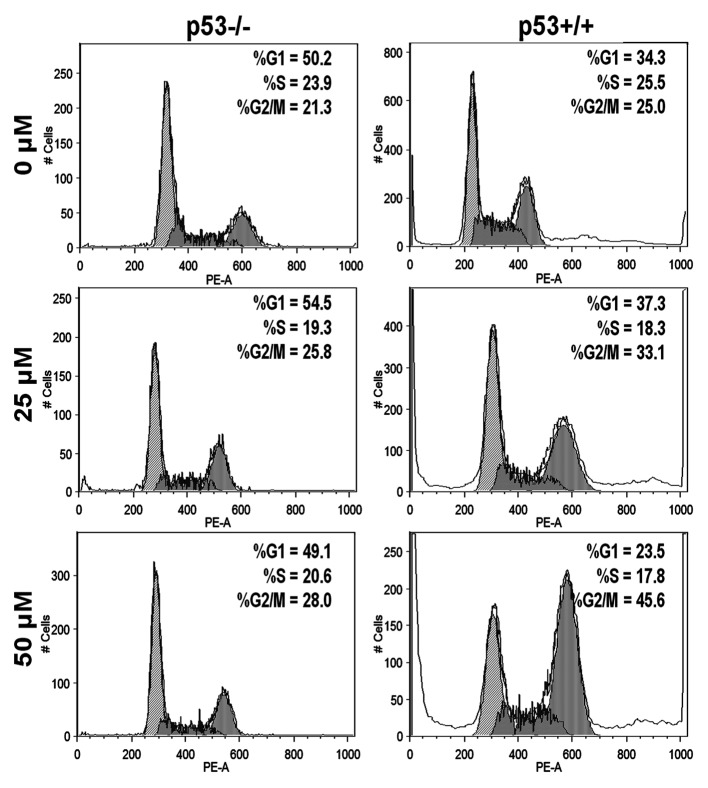
Genistein induces G2/M cell cycle arrest in both HCT-116 p53^+/+^ and p53^−/−^ cells via a p53-dependent pathway. Genistein arrested more HCT-116 p53^+/+^ cells in the G2/M phase than in the p53^−/−^ cells at concentrations (25 and 50 *μ*M) (33.1 vs 25.8%, 45.6 vs 28%).

To evaluate whether p53 is a key factor in G2/M cell arrest induced by genistein, we performed a cell cycle assay to investigate both HCT-116 p53^+/+^ and p53^−/−^ cells. Fig. 5 shows that genistein arrested more p53^+/+^ cells in the G2/M phase than p53^−/−^ cells at 25 and 50 *μ*M (p<0.01, statistically determined by 3 independent experiments), which suggests that p53 plays a pivotal role in promoting G2/M cell cycle capture in the HCT-116 cell line.

### Genistein enhances ATM/p53-p21 expression and suppresses cdc2, cdc25A expression in p53^+/+^ HCT-116 cells

**Figure 6.**
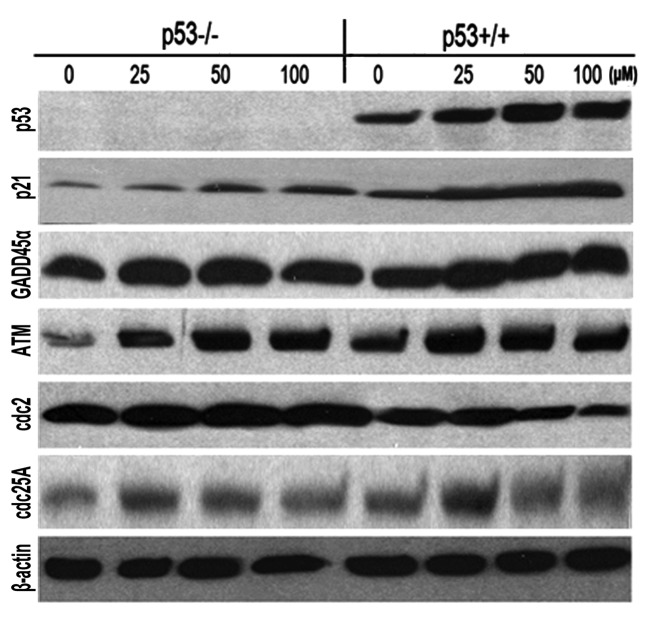
Genistein induces G2/M phase arrest via p53/ATM-p21*^waf1/cip1^* activation and cdc2/cdc25A downregulation. Western blotting of p53, p21, GADD45α, ATM, cdc2, cdc25A and β-actin in HCT-116 p53^+/+^ and p53^−/−^ cells were taken after treatment of genistein at the indicated doses (25, 50 and 100 *μ*M).

HCT-116 p53^+/+^ and p53^−/−^ cells were exposed to different concentrations of genistein (25, 50, 100 *μ*M) for 72 h and the expression of p53 and its targets, p21*^waf1/cip1^*, GADD45α, were determined by western blotting. β-actin was used as a control. p53 increased in a dose-dependent manner in HCT-116 p53^+/+^ cells (Fig. 6). The expression of p21*^waf1/cip1^* and GADD45α was enhanced in both HCT-116 p53^+/+^ and p53^−/−^ cells (Fig. 6). It should be noted that p21*^waf1/cip1^* was increased notably in p53^+/+^ cells compared to p53^−/−^ cells, which suggested that p53 is an important transcriptional regulator of p21*^waf1/cip^*. Ataxia telangiectasia mutated (ATM), which acted as an upstream regulator and co-regulator of p53 in cell cycle processing, also increased along with p53 and p21*^waf1/cip1^* in both cell types. cdc2 and cdc25A, the effectors of p53/p21*^waf1/cip1^*, were downregulated in our experiments. These two molecules dramatically deceased in p53^+/+^ cells compared with the p53^−/−^ cells, which might contribute to the fact that genistein induced a stronger cell cycle capture effect in HCT-116 cells with a stable p53 status.

## Discussion

Plant-derived polyphenolic compounds containing isoflavones have attracted considerable interest for their anticancer properties. However, the mechanism of their anticancer effects remains to be fully understood. In this study, we showed that the soy isoflavones genistein, daidzein and biochanin A had significant inhibitory effects on cell growth and promoted apoptosis in human colon cancer cells. Among them, genistein was the most attractive agent for G2/M phase cell cycle arrest. As one of the major components of soy isoflavones, genistein clearly exhibited anticancer effects in a time- and dose-dependent manner, which might probably make it a competitive candidate for anticancer research. Furthermore, we observed that genistein activated the ATM/p53-p21*^waf1/cip1^* cross-talk regulatory network, a pathway implicated in G2/M cell arrest and apoptosis. A microarray-based qPCR array presented that several genes involved in cell cycle and apoptosis, such as CDKN1b, BRCA1 and BRCA2 and Bcl2, were examined and underwent transcriptional activation after genistein exposure. This suggests that genistein regulates multiple regulatory factors, including the cyclin families and Bcl families, to modulate the complicated cancer development and progress.

Kim *et al* reported that genistein and daidzein suppressed cell proliferation and induced apoptosis in HT-29 cells and that these events were related to the inhibition of insulin-like growth factor-1 (IGF-1) receptor signaling and the PI3k/AKT pathway, also including the involvement of the MAPK pathway (20). The MAPK pathway, which includes ERK-1/2 and AKT as key regulators, has been reported to be crucial in cell proliferation and growth (21). Specifically, the ERK-1/2 signaling pathway has been implicated in the regulation of cell cycle phases such as G1 and G2/M. With regard to colon cancer, soy isoflavones have been shown to inhibit HT-29 cell (22) and Colo320 cell (23) growth by an accumulation of cells at the G2/M phase. p53, acting as an important tumor suppressor gene, regulates cancer cell progression through multiple mechanisms, including the induction of apoptosis and cell cycle arrest, which has been well reviewed (24). It is encouraging that, consistent with previous studies, we observed that genistein exerted anticancer effects partially in a p53-dependent manner. Genistein arrested HCT-116 cells at the G2/M phase in a dose-dependent manner. This was correlated to its effect of triggering the upregulation of a tumor suppressor gene, p53, as well as the CDK inhibitor p21*^waf1/cip1^*. The results are further substantiated by the accompanied decreases in the expression of the cyclin family complex proteins, cdc2 and cdc25A. p53 status is crucial to the apoptosis and cell cycle arrest on human colorectal cancer cell line HCT-116.

It should be noted that genistein did not increase the expression of p27*^kip1^* by q-PCR assay in our study, which implied that p21*^waf1/cip1^* played a more important factor in G2/M cell capture induced by genistein exposure. A recent report suggested that genistein inhibited EGF-induced proliferation, which favors dephosphorylation and nuclear retention of FOXO3 in colon cancer cells. Upstream of FOXO3, genistein acts via the PI3K/Akt pathway to inhibit EGF-stimulated FOXO3 phosphorylation. Downstream, EGF induced disassociation of FOXO3 from mutated tumor suppressor p53, but not wild-type p53, which is inhibited by genistein favoring FOXO3-p53(mut) interactions with the promoter of the cell cycle inhibitor p27*^kip1^* in colon cancer cells. The FOXO3-p53(mut) complex leads to elevated p27*^kip1^* expression and promotes cell cycle arrest (25). This might be interpreted that the partially p53-dependent cell cycle has an arresting mechanism in p53-wild-type or p53-mutanted human cancer cell lines. Our experiments mainly aimed at studying the contributions of p53 on G2/M cell arrest. Other pathways involved in the anticancer properties of the compounds, including those that mediate cell death and apoptosis, are still in need of further investigation.

Eukaryotic cells employ multiple mechanisms to ensure accurate transmission of genetic information between generations. Critical surveillance of this transmission is provided by the DNA damage response (DDR) (26). Disruption or attenuation of DDR plays an essential role in promoting tumorigenesis (27–29). This system not only functions in the process of DNA damage repair, but is also integrated with other processes including the cell cycle, apoptosis and transcription (26). DNA damage was able to activate the MEK-ERK pathway in a p53-dependent (30) and independent manner (31). In our study, genistein induced G2/M cell arrest via the p53/ATM pathway in a dependent way, which suggested that p53 has a more crucial role in controlling cell expansion. As demonstrated by numerous studies, p53 is a key tumor suppressor gene and is one of the most important mainstays of our body’s anticancer defense (32). In response to multiple cellular stresses, such as DNA damage and hyperproliferation (18,33), p53 is activated or stabilized. Once induced, p53 can function as a transcription factor to regulate the several genes leading to apoptosis, senescence and cell cycle arrest (34–36). In the early stages of tumor development, genomic instability and DNA damage lead to p53 activation and mediate tumor suppression (37,38). Our data showed that ATM/p53-p21*^waf1/cip1^* was activated when human colon cancer cells were treated by genistein. Similar to previous studies, genistein exposure introduced DNA damage or genotoxic stress, which could lead to activation of ATM and subsequently induce p53 expression. Through phosphorylation, p53 induces the expression of p21*^waf/cip1^* as well as GADD45α. Accordingly, cdc2 and cdc25A activity is inhibited, which results in the capture of G2/M phase cells. The G2/M checkpoint prevents cells from initiating mitosis in the presence of DNA damage (26,39). Interestingly, p53 was regarded as the most important regulatory factor in the G2/M cell cycle checkpoint, but we observed that it is not the only triggering factor for controlling cell cycle transit, which suggests that a complicated regulatory network is involved in the cell cycle progression in response to DNA damage in HCT-116 p53 wild or p53 mutant cells. Our findings presented here suggest that p53-p21*^waf1/cip1^*/cdc2 cross-talk co-regulates cell cycle distribution at different phases, which could possibly be an important mechanism for understanding the cell cycle progress induced by genistein in human colon malignancies.

Taken together, our data showed that isoflavones, such as genistein, inhibit human colon cell proliferation and growth by causing cell arrest at the G2/M phase and promoting substantial apoptosis. These processes are primarily mediated by the upregulation of p53/p21*^waf1/cip1^*, GADD45α and down-regulation of cdc2 and cdc25A. In response to DNA damage, the cells trigger the checkpoint signaling cascades to regulate cell cycle progression and elicit DNA repair mechanisms in need of maintaining genomic stability and integrity. High doses of genistein not only induce DNA damage and block the cell cycle, but they also initiate programmed cell death. These findings may provide some new insights into the genotoxic effects and antitumor mechanisms of genistein and other isoflavonoids in human colon cancers.
